# Supplementary figures and images for: Modeling target-density-based cull strategies to contain foot-and-mouth disease outbreaks
Source: PeerJ. 2024 Feb 29;12:e16998. doi: 10.7717/peerj.16998 (PMC10909358; doi:10.7717/peerj.16998)

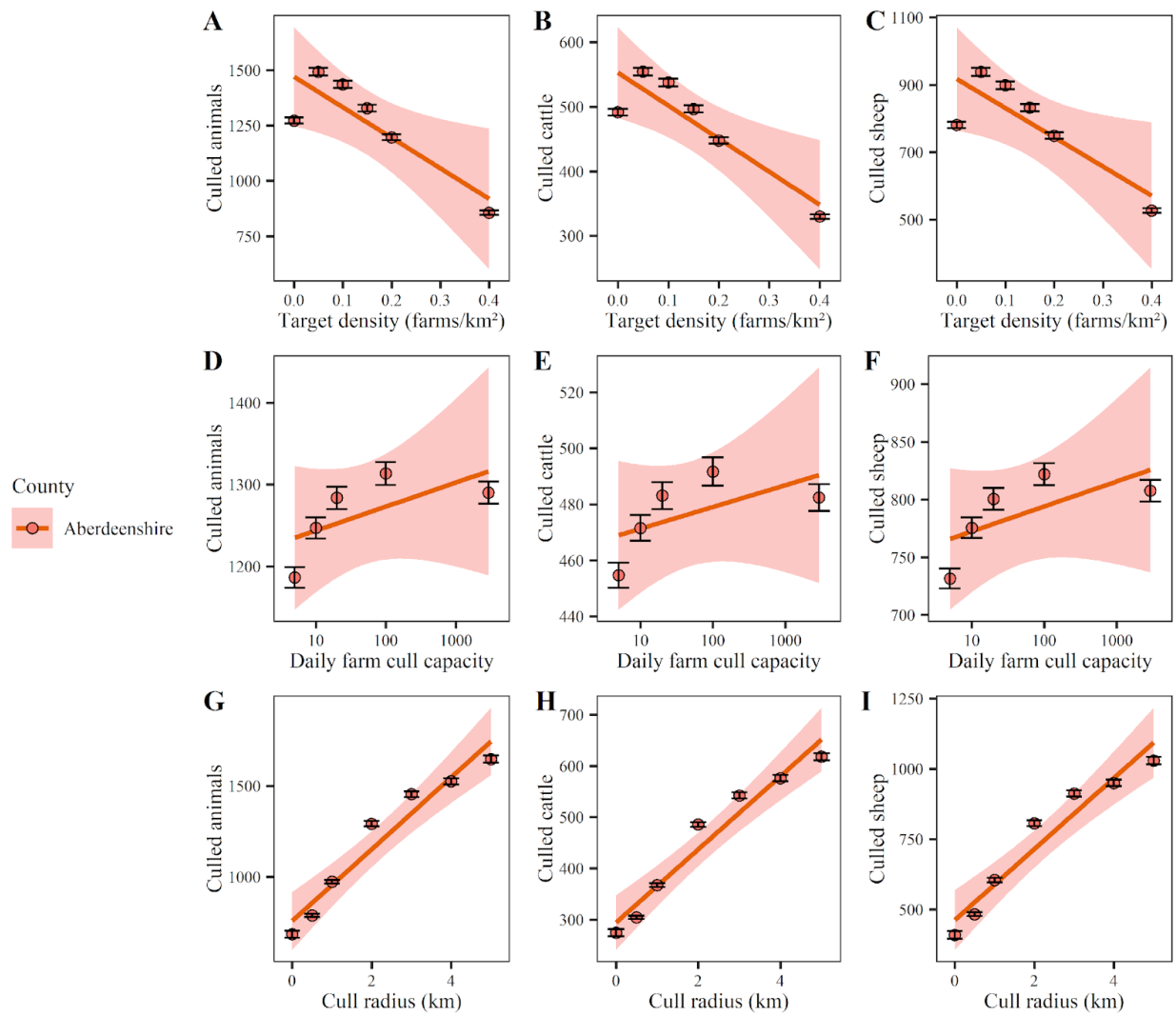

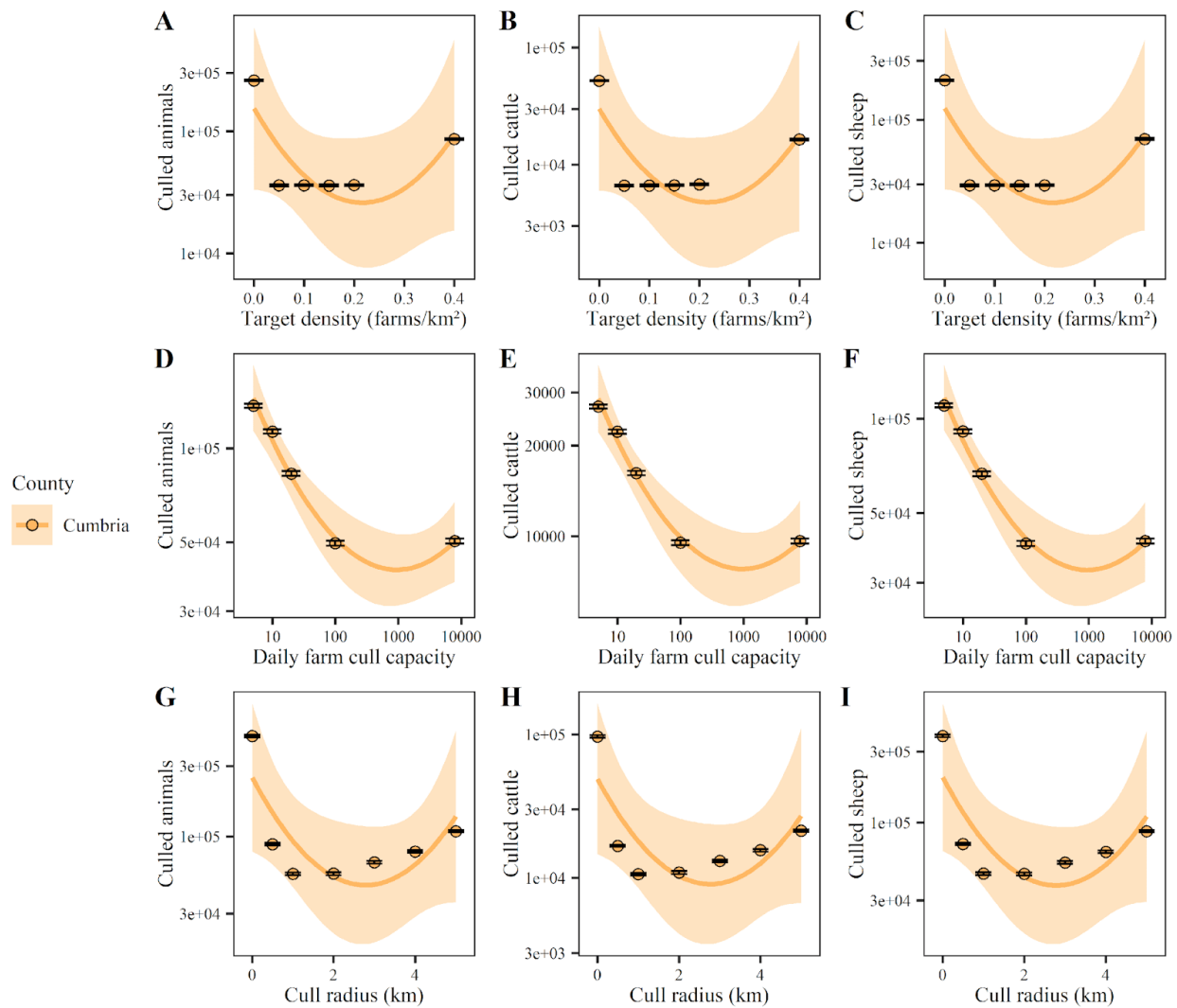

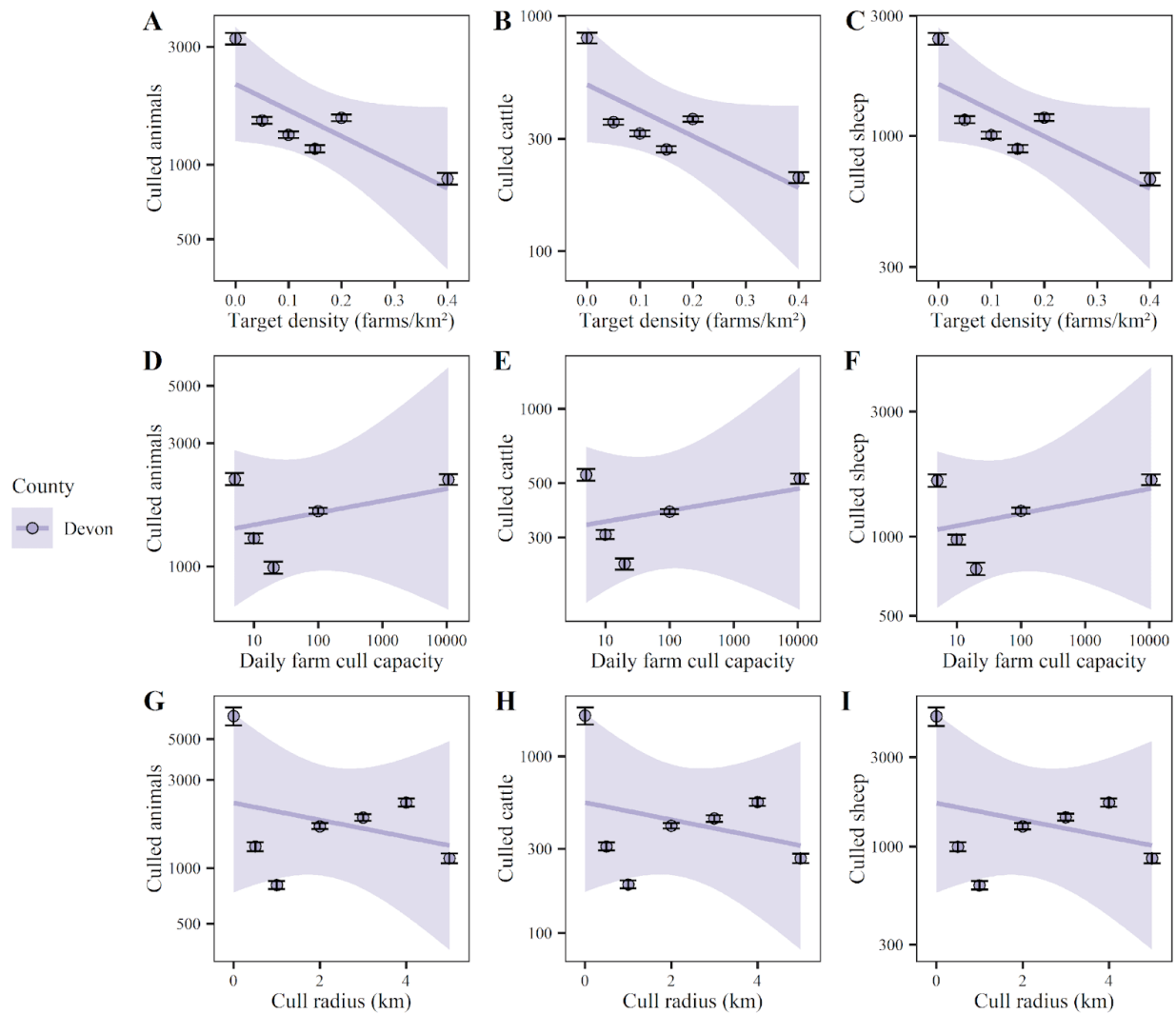

County  
North Yorkshire

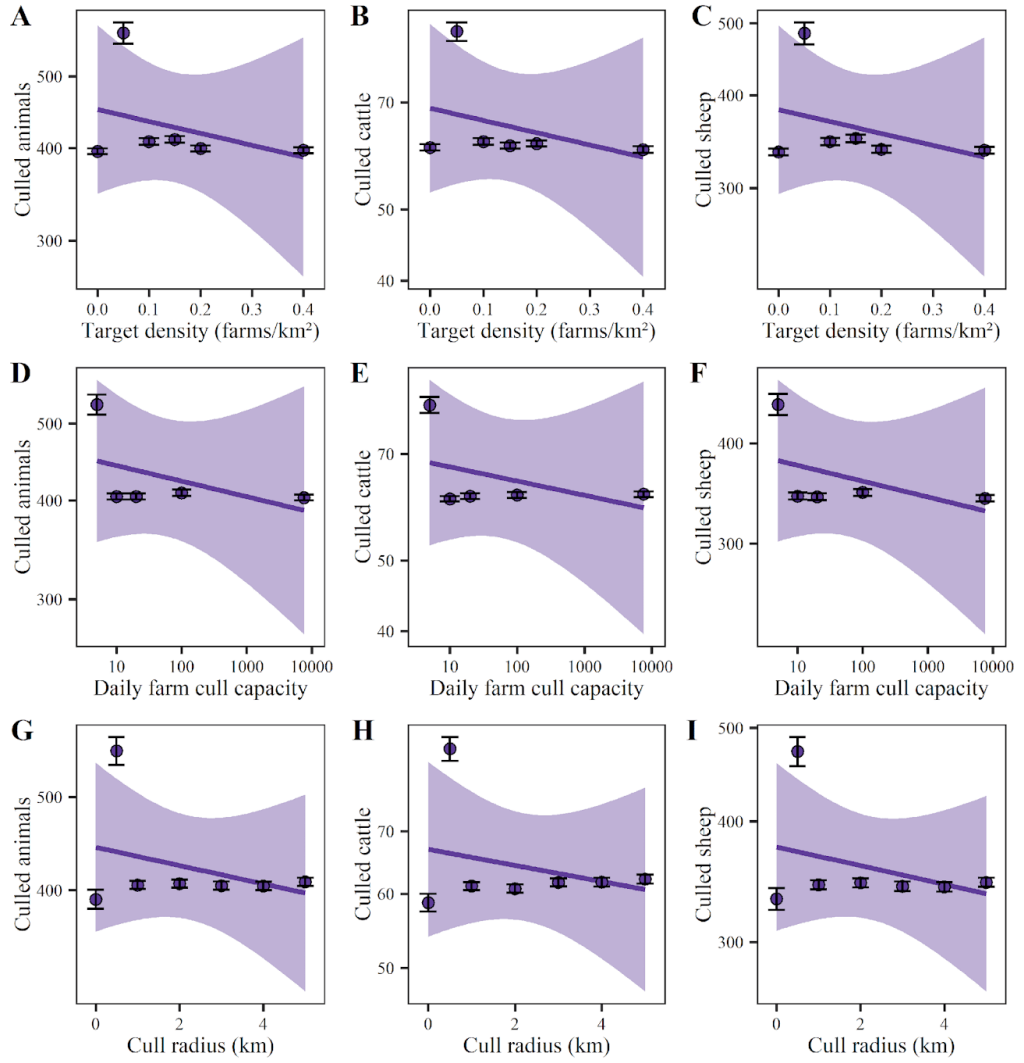

Supplement: Supplemental Information 1 — The four counties are shown in panels of different color. Target size culling implementation variables are on the x-axes and include target farm cull density, daily farm cull capacity, and cull radius. Farm response variables are on the y-axes and include total culled animals (cattle and sheep) per county, total culled cattle per county, and total culled sheep per county. The points and error bars indicate means ± standard errors. The curves are quadratic regressions fit to the means and shaded areas are standard errors of the regressions. (A–) The y-axis is log10-transformed; (D–F) The x-axis is log10-transformed. Each data point is the mean over 1,000 simulations. [file peerj-12-16998-s001.pdf]

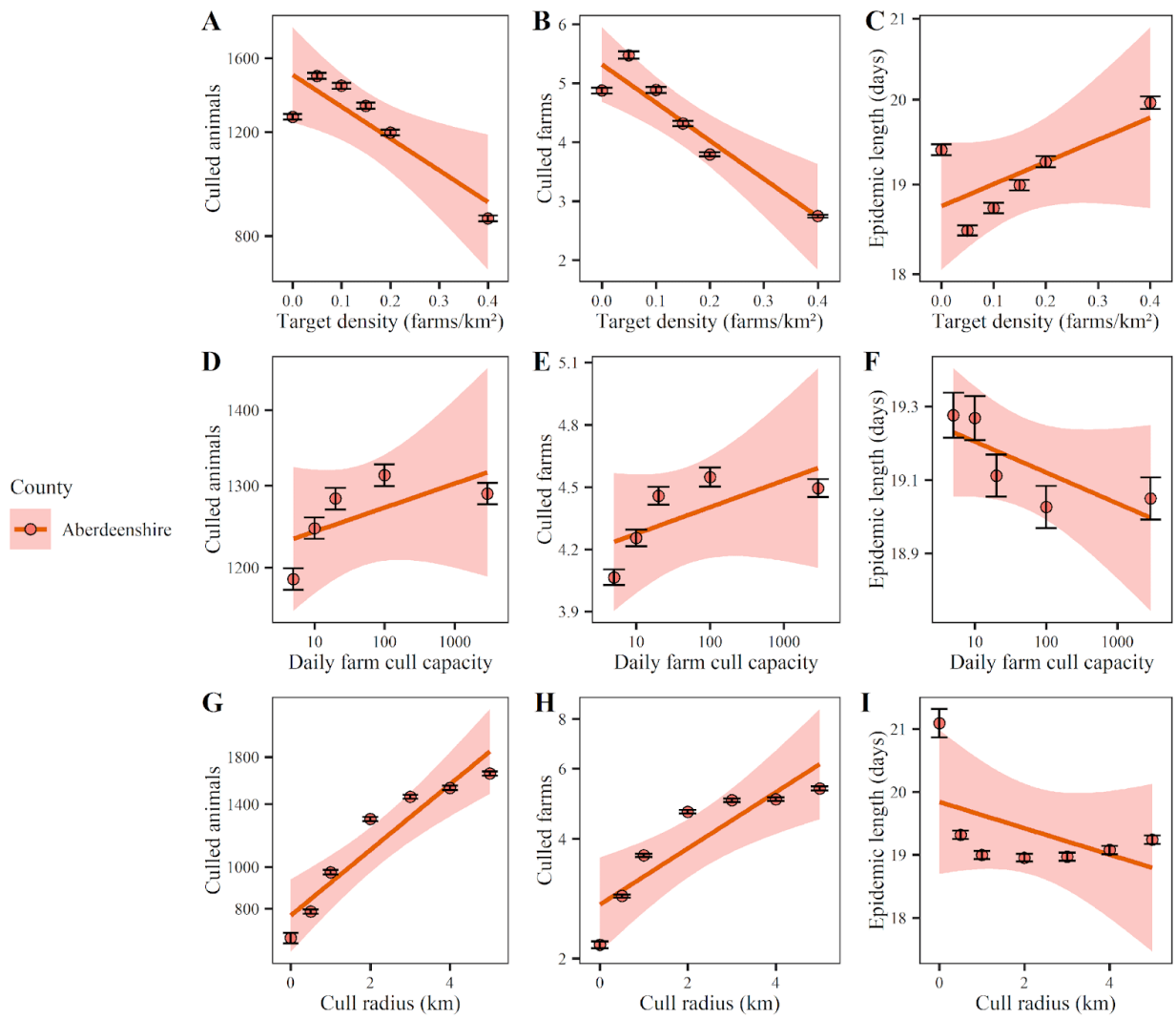

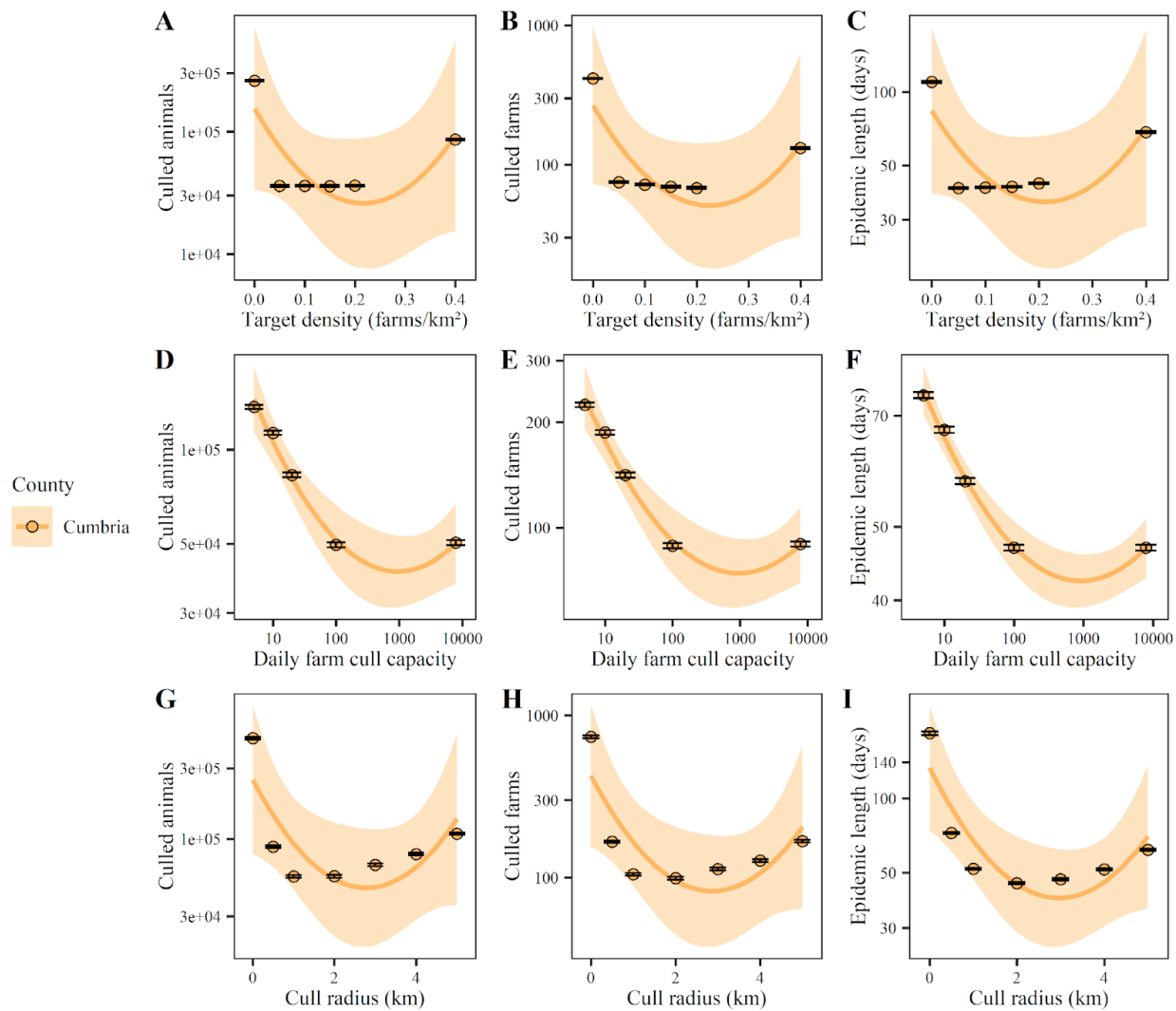

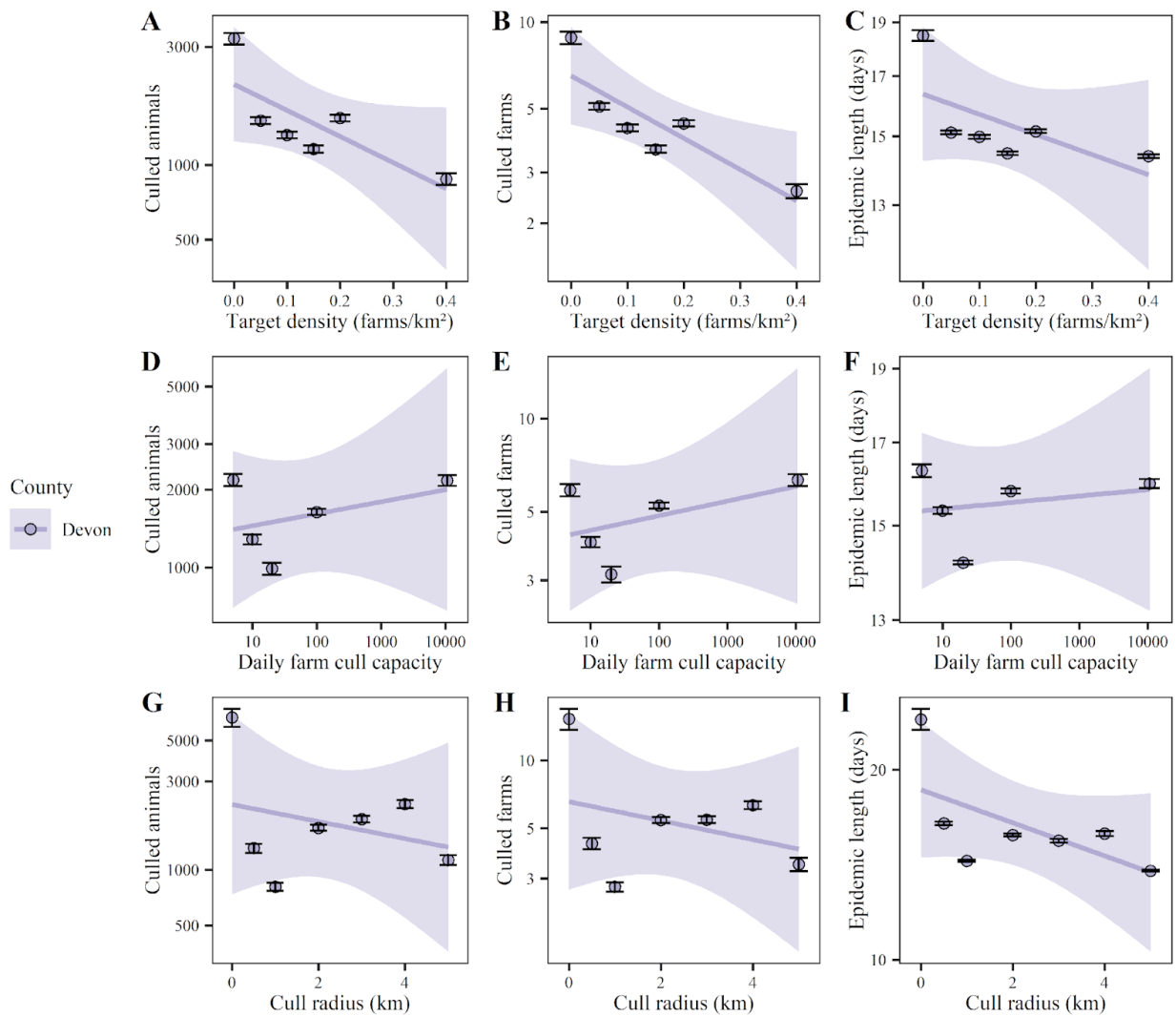

County  
North Yorkshire

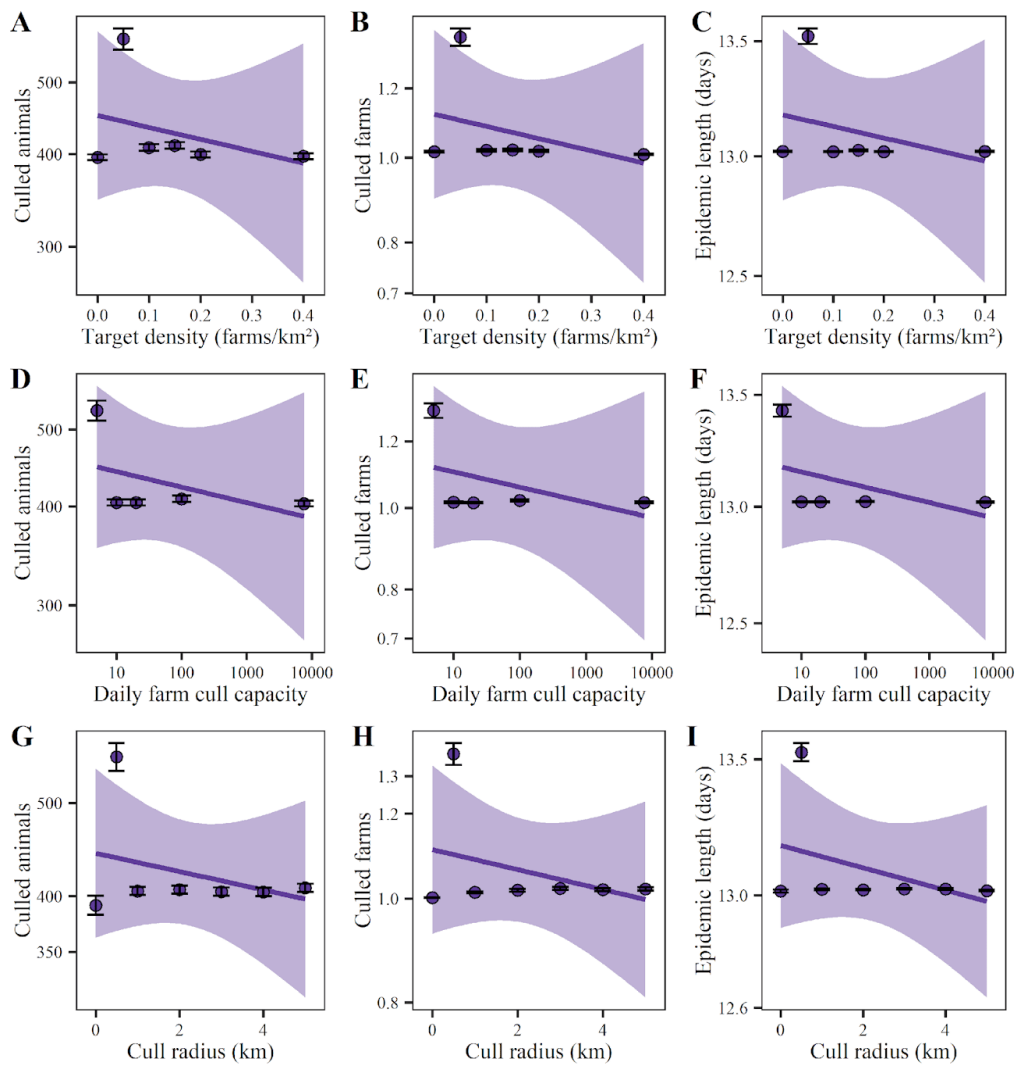

Supplement: Supplemental Information 2 — The four counties are shown in panels of different color. Target size culling implementation variables are on the x-axes and include target farm cull density, daily farm cull capacity, and cull radius. Farm response variables are on the y-axes and include total culled animals (cattle and sheep) per county, total culled cattle farms per county, and mean epidemic length per county. The points and error bars indicate means ± standard errors. The curves are quadratic regressions fit to the means and shaded areas are standard errors of the regressions. (A–I) The y-axis is log10-transformed; (D–F) The x-axis is log10-transformed. Each data point is the mean over 1,000 simulations. [file peerj-12-16998-s002.pdf]

County  
Aberdeenshire

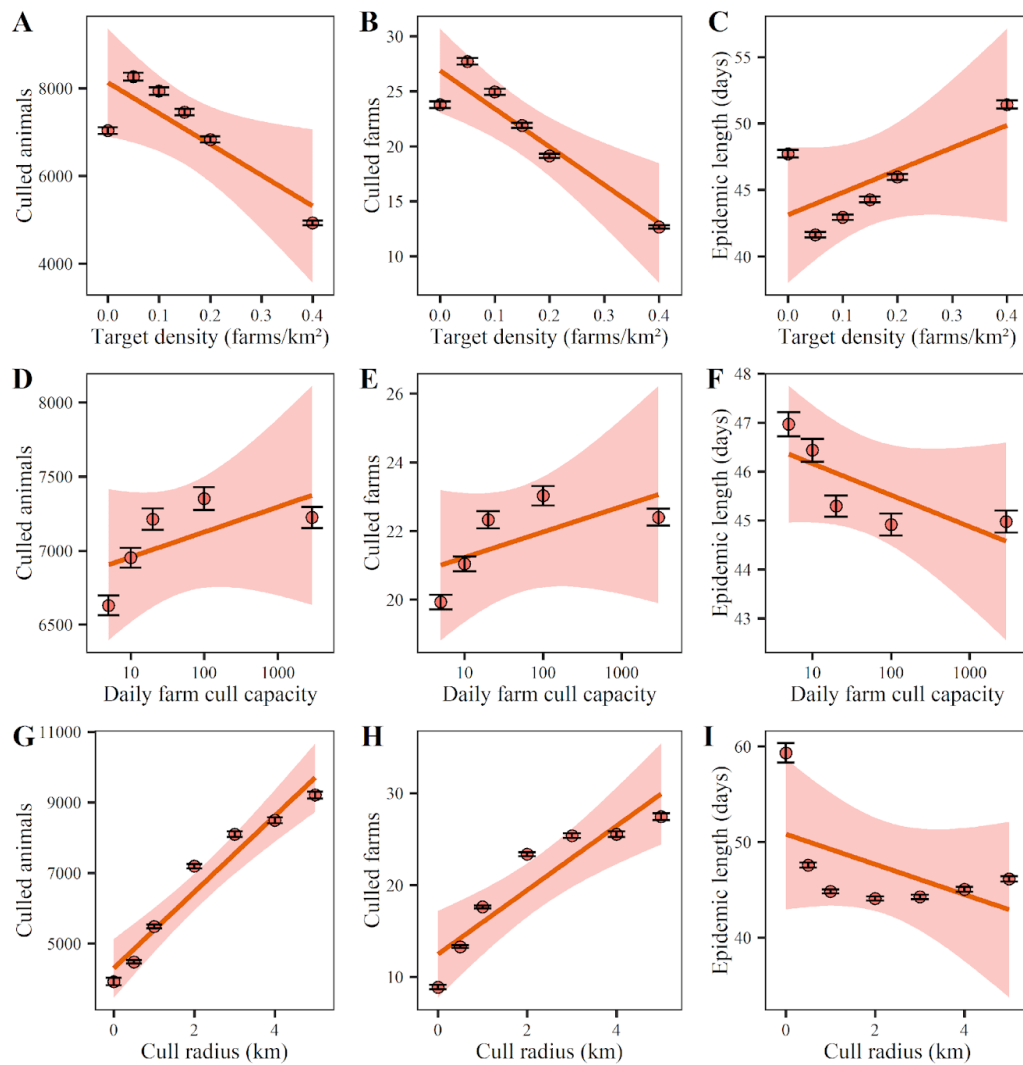

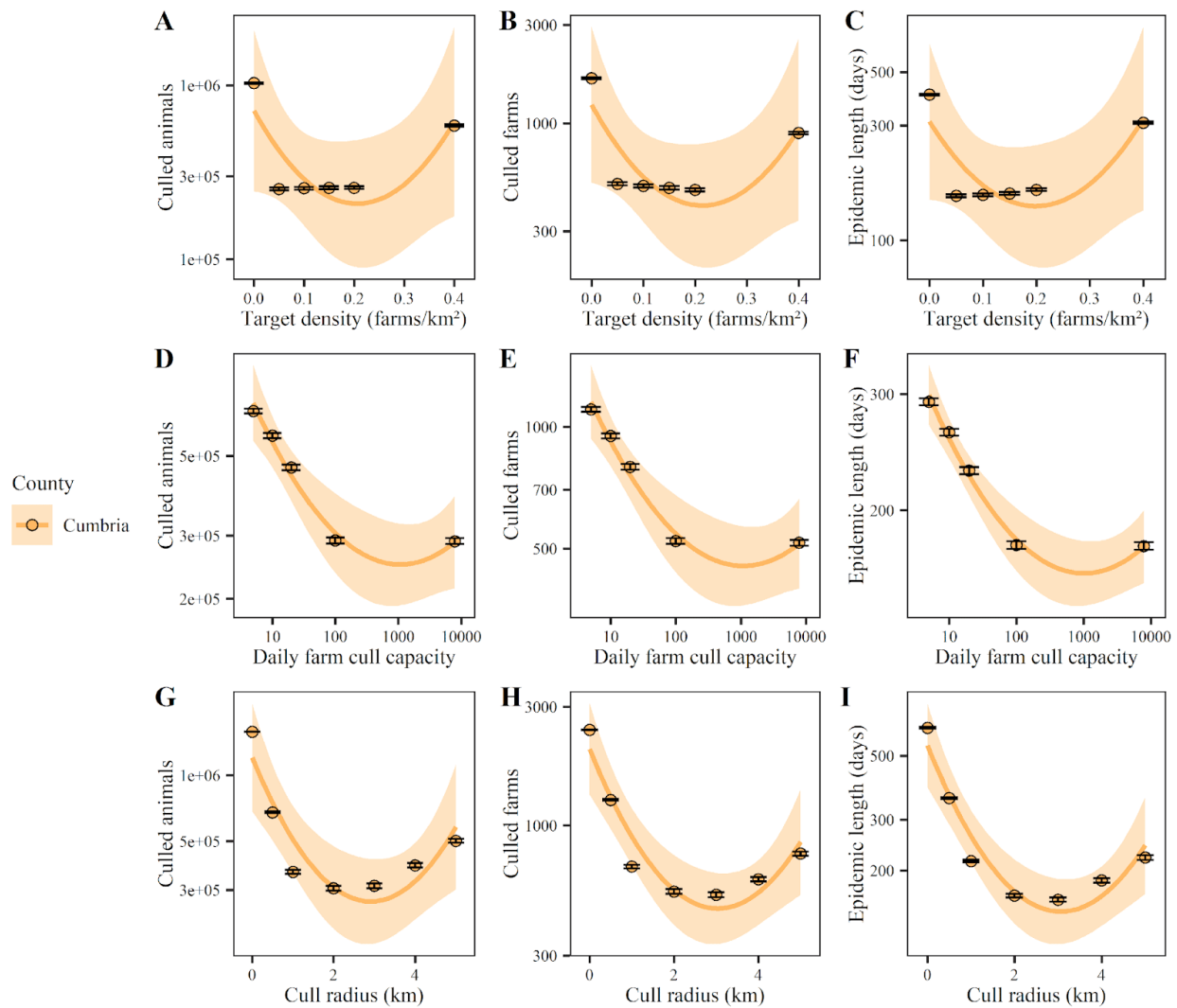

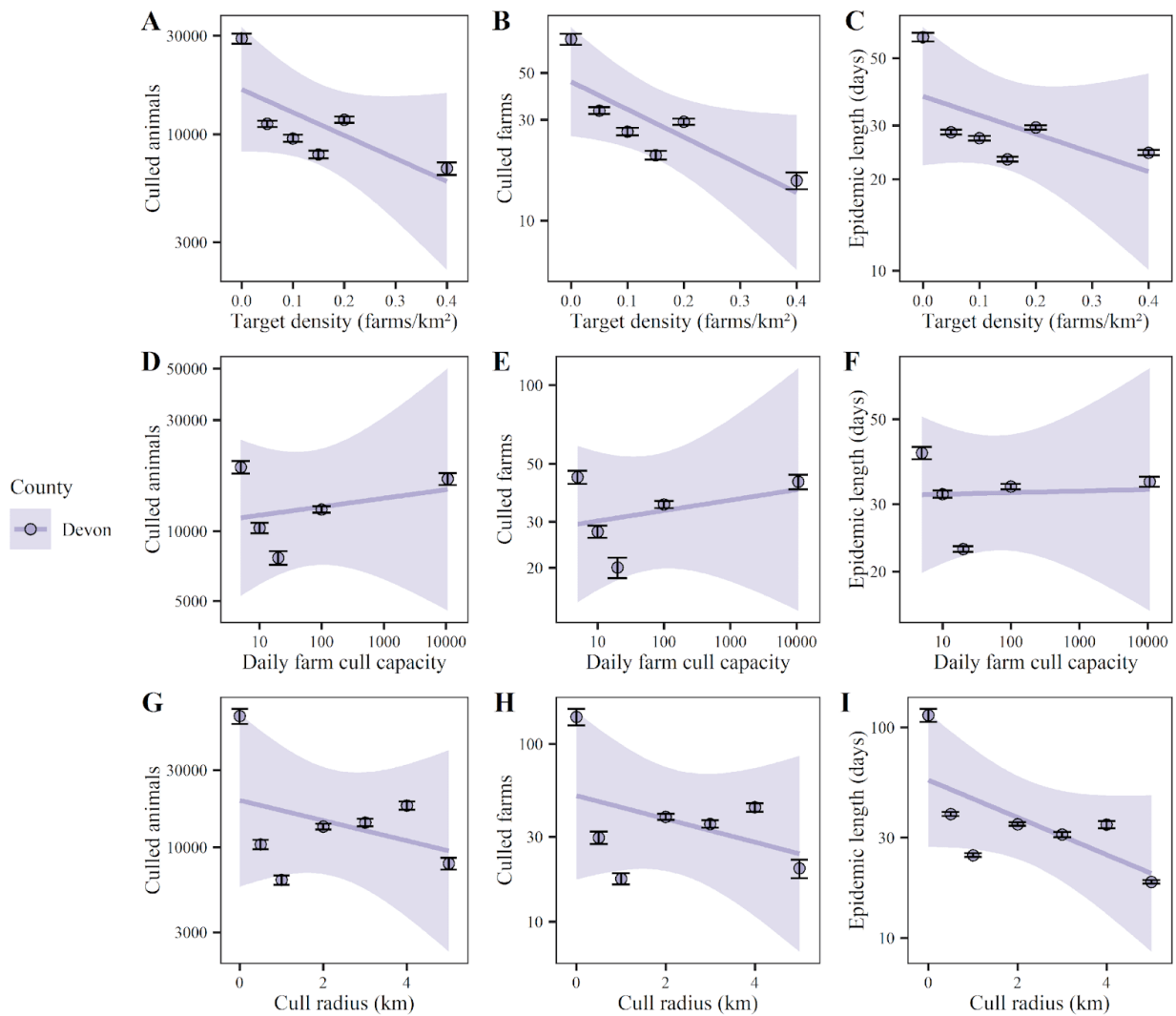

County  
North Yorkshire

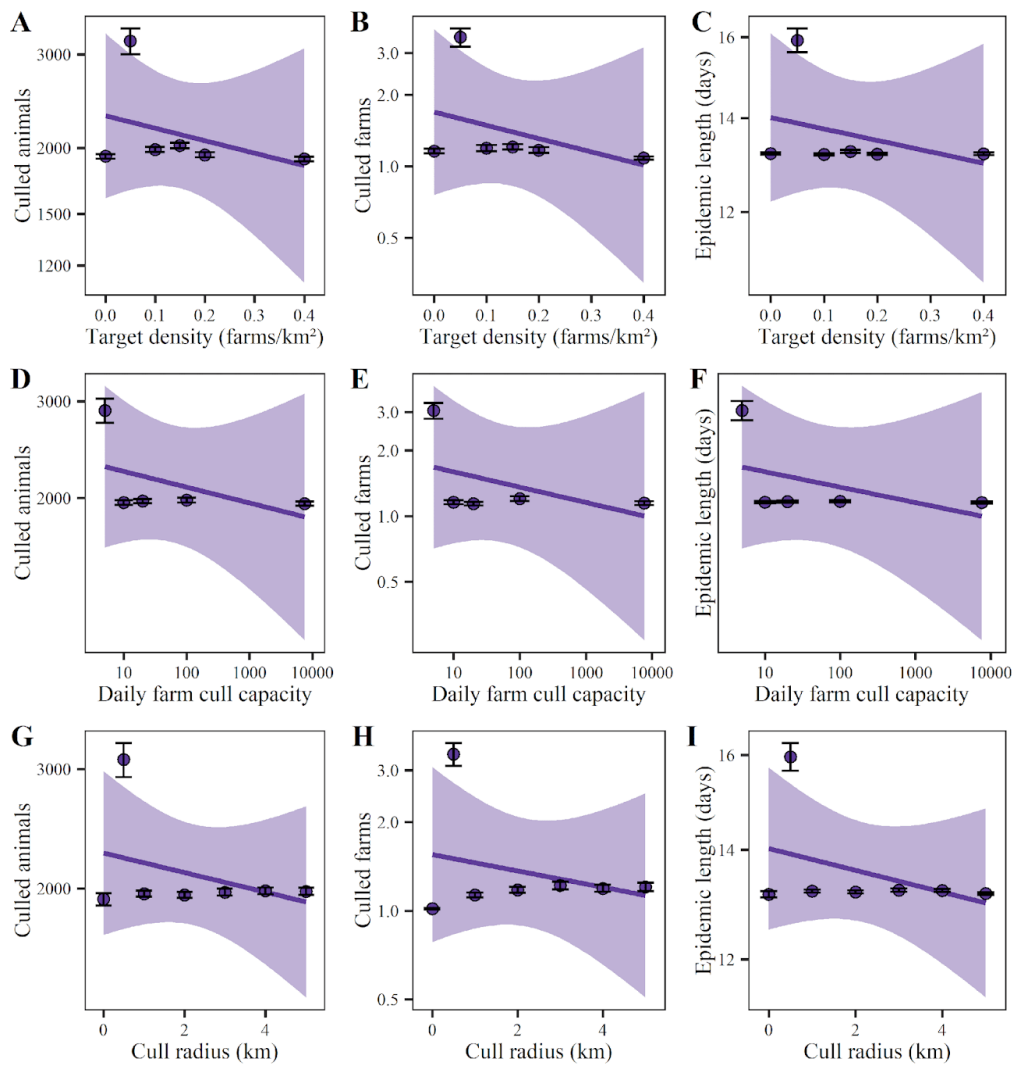

Supplement: Supplemental Information 3 — The four counties are shown in panels of different color. Target size culling implementation variables are on the x-axes and include target farm cull density, daily farm cull capacity, and cull radius. Farm response variables are on the y-axes and include total culled animals (cattle and sheep) per county, total culled cattle farms per county, and mean epidemic length per county. The points and error bars indicate means ± standard errors. The curves are quadratic regressions fit to the means and shaded areas are standard errors of the regressions. (A–I) The y-axis is log10-transformed; (D–F) The x-axis is log10-transformed. Each data point is the mean over the 10% most severe epidemics for each epidemic impact (culled animals, culled farms, and epidemic length. [file peerj-12-16998-s003.pdf]

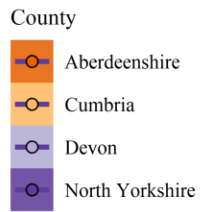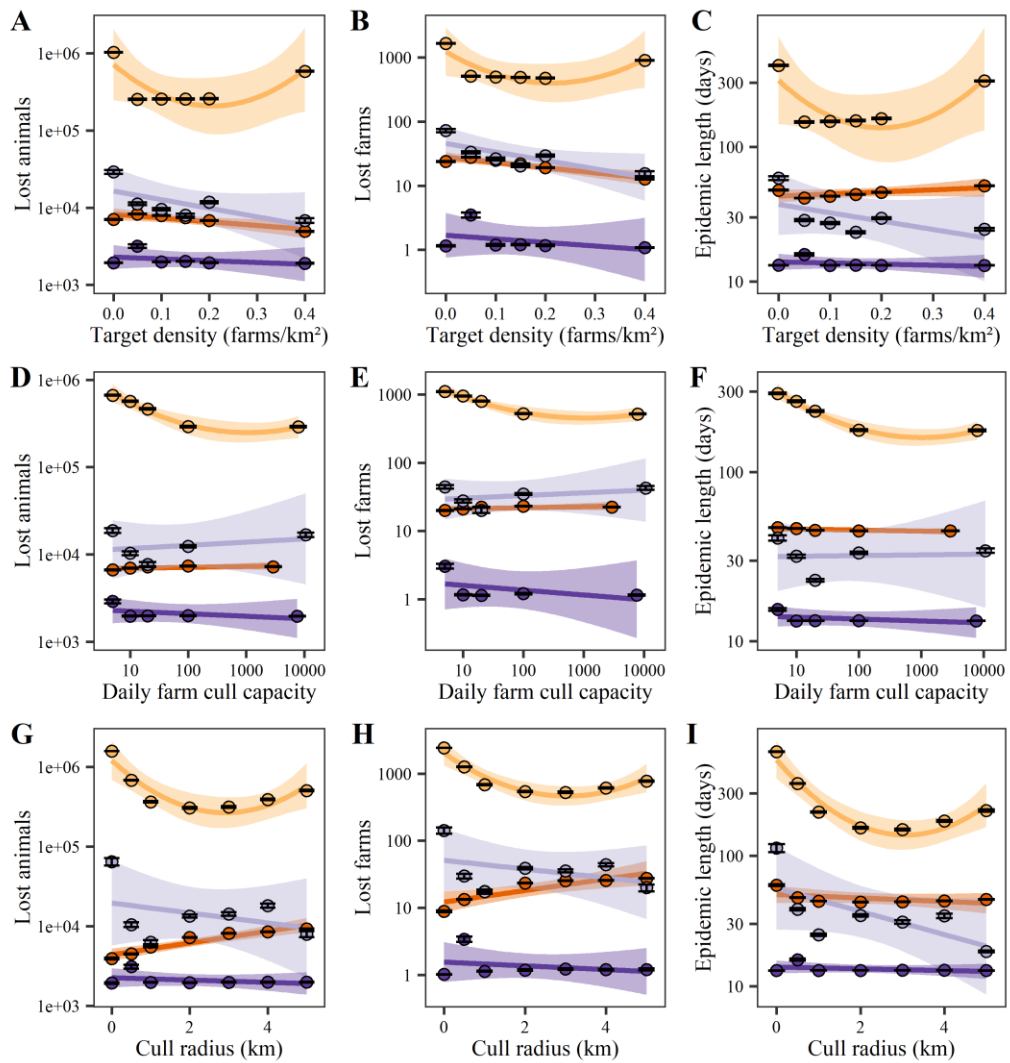

Supplement: Supplemental Information 4 — (A) target farm cull density and culled animals, (B) target farm cull density and culled farms, (C) target farm cull density and epidemic length, (D) daily farm cull capacity and culled animals, (E) daily farm cull capacity and culled farms, (F) daily farm cull capacity and epidemic length, (G) cull radius and culled animals, (H) cull radius and culled farms, (I) cull radius and epidemic length. The points indicate means over 100 simulations (the top 10 percent of each epidemic impact measure, i.e., culled animals, culled farms, and epidemic length. The curves and shaded regions are quadratic regressions fit to the data by county ± standard error. The different colors and shapes indicate different counties. (A–I) The y-axis is log10-transformed. (D–F) The x-axis is log10-transformed. [file peerj-12-16998-s004.pdf]

A)

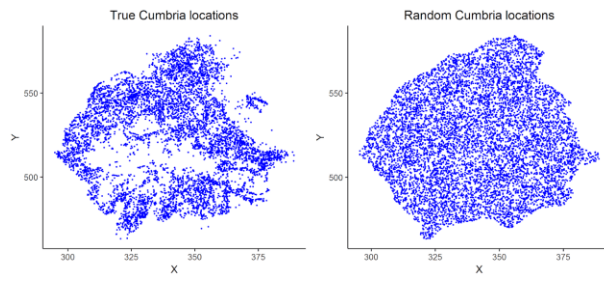

B)

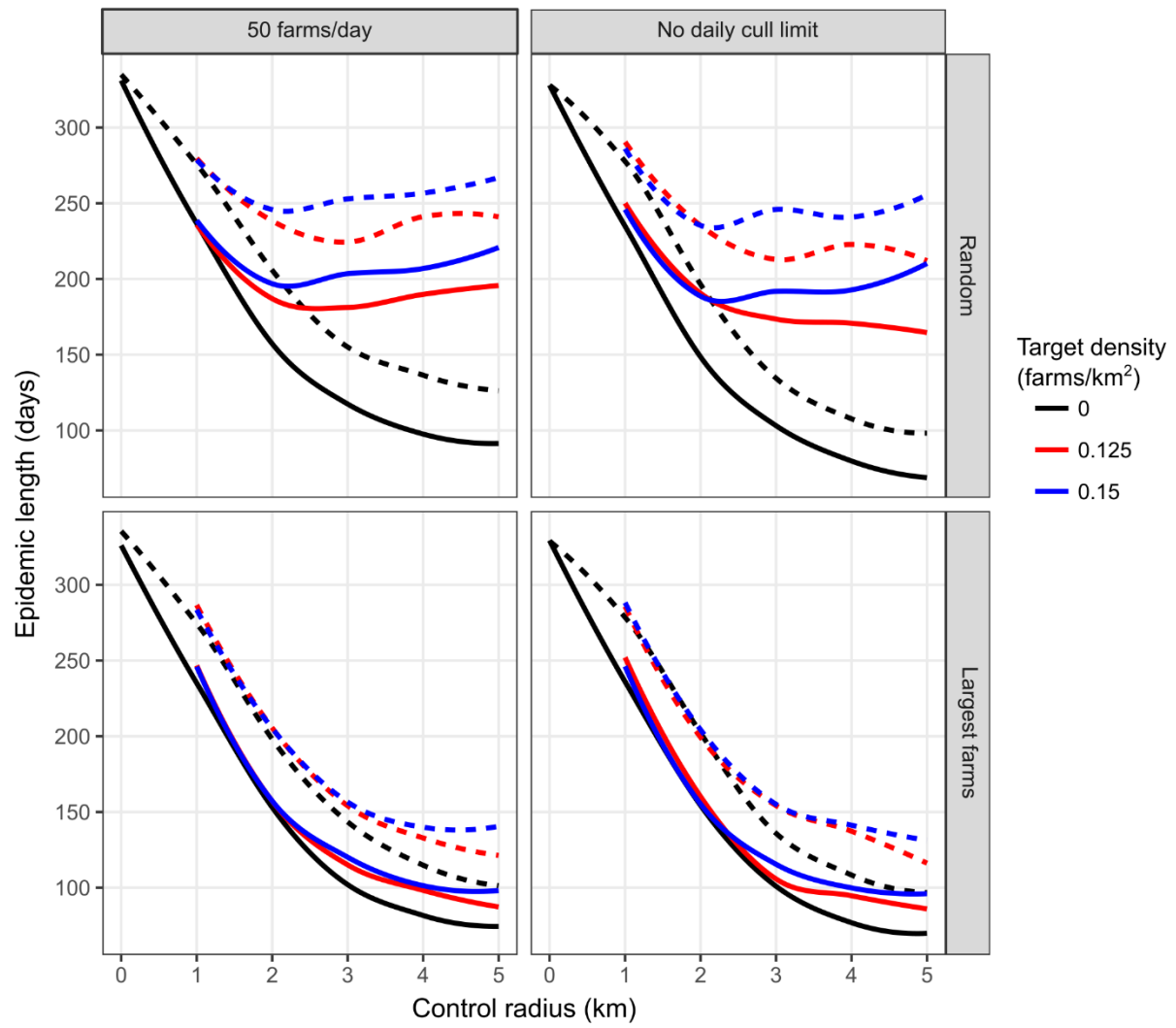

Supplement: Supplemental Information 5 — (A) FMD outbreaks were simulated using true and random Cumbria farm locations, indicated by points. (B) Lengths of FMD epidemics are shown for true (dashed lines) and random (solid lines) of Cumbria farm locations. Infected farms were located and culled to achieve the target farm density within the control radius. Each panel shows a unique combination of daily cull capacity (50 farms/day or unrestricted; columns) and prioritization for culling farms within the control radius (randomly select farms or target the largest farms; rows). [file peerj-12-16998-s005.pdf]

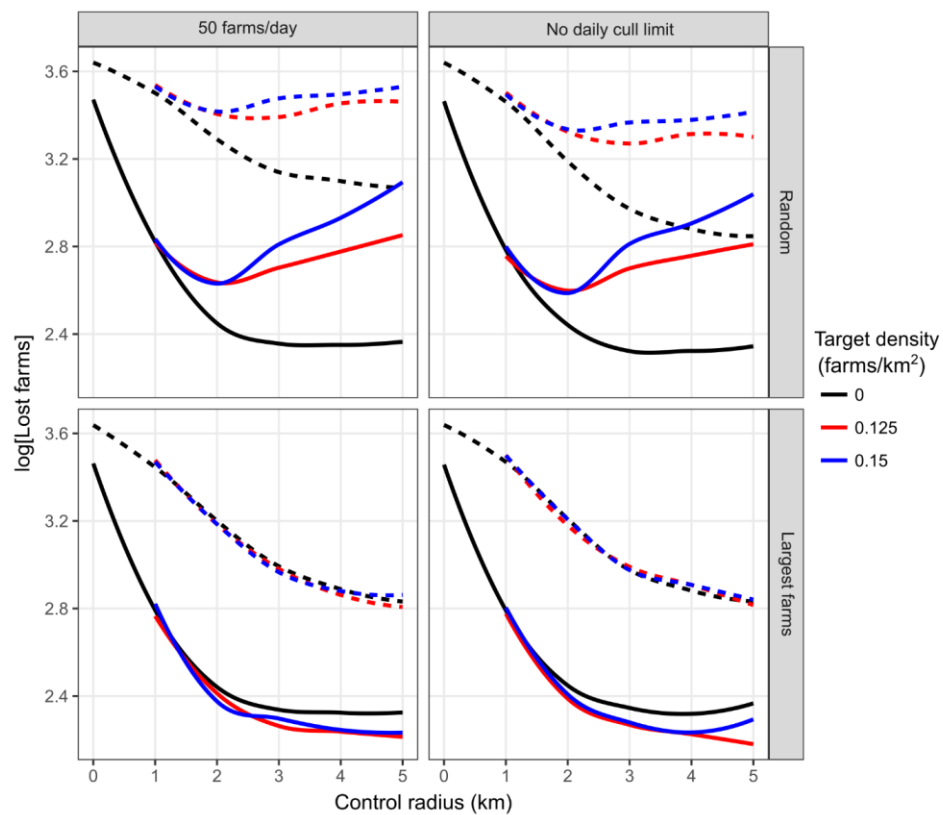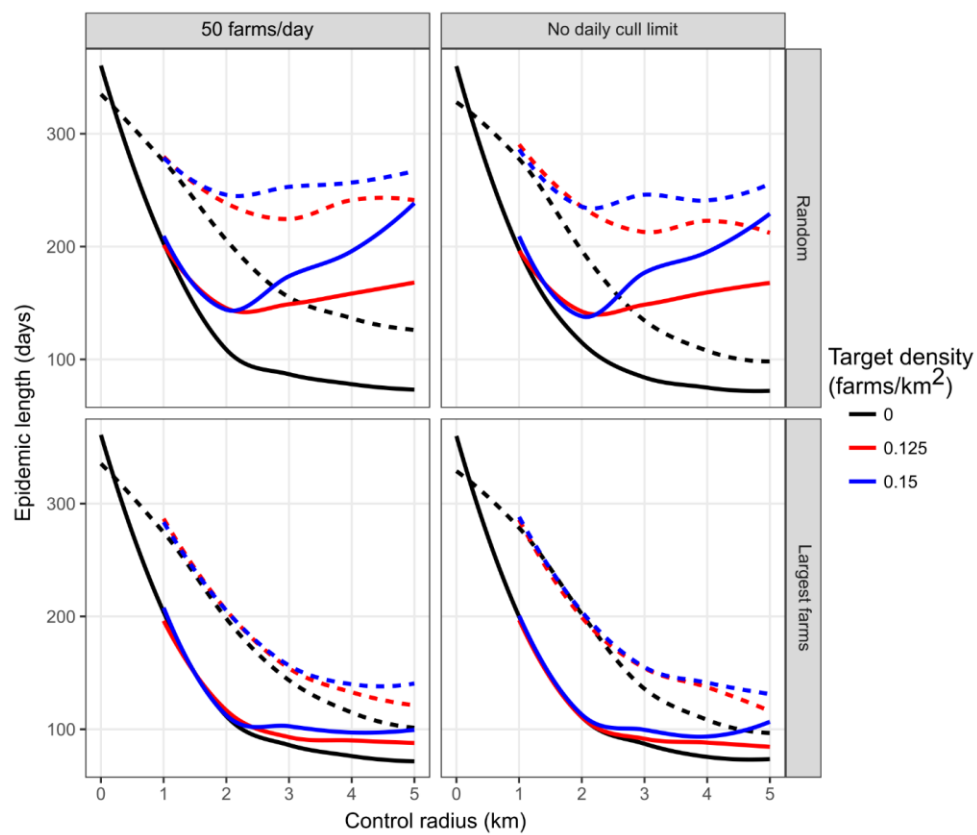

Supplement: Supplemental Information 6 — Total epidemic impact includes number of farms lost to both infection and control methods, upper four panels) and epidemic length (lower four panels). Susceptible farms located within the control radius of an IP were culled to achieve the target farm density (color scale) within the control radius. Each panel shows a unique combination of daily cull capacity (50 farms/day or unrestricted; columns) and prioritization strategy for culling farms within the control radius (randomly select farms or target the largest farms; rows). [file peerj-12-16998-s006.pdf]

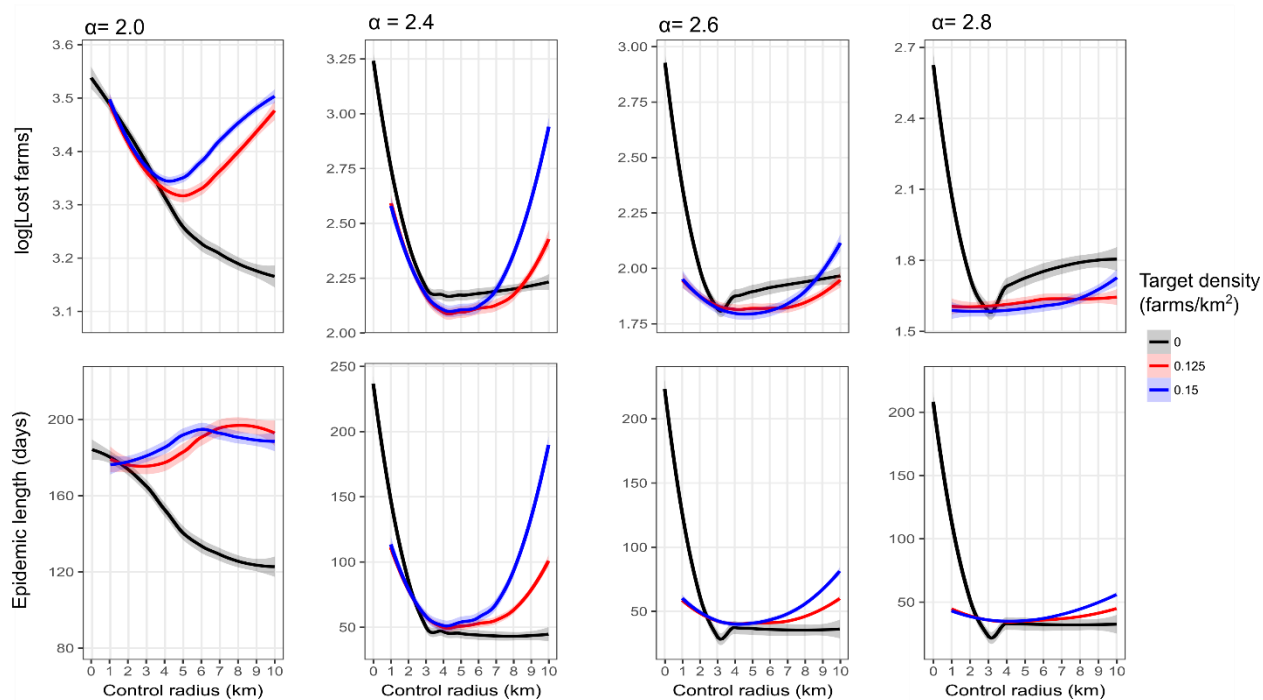

Supplement: Supplemental Information 7 — Results shown are the mean epidemic impact (lines) and 95% C.I. (shaded regions) for each target density cull strategy (color scale). The value at 0 km control radius represents the ‘stamping-out’ control strategy. Each column of panels show results for the indicated value of the kernel shape parameter when the daily cull limit is 50 farms/day and the largest farms within control radii are prioritized for cull. Each data point is the mean over 1,000 simulations. [file peerj-12-16998-s007.pdf]
