# Supplementary material for: Modeling target-density-based cull strategies to contain foot-and-mouth disease outbreaks
Source: PeerJ. 2024 Feb 29;12:e16998. doi: 10.7717/peerj.16998 (PMC10909358; doi:10.7717/peerj.16998)
Supplement: Supplemental Information 8 — The statistics include number of simulations (N), mean, standard deviation (sd), standard error (se), and 95% confidence interval (ci). [file peerj-12-16998-s008.pdf]

Culled cattle by target density after culling

County	Density (farms/km^2)	N	Mean culled cattle	sd	se	ci
Aberdeenshire	0	35000	491	942	5	10
Aberdeenshire	0.05	30000	554	1048	6	12
Aberdeenshire	0.1	30000	538	1020	6	12
Aberdeenshire	0.15	30000	497	932	5	11
Aberdeenshire	0.2	30000	448	837	5	9
Aberdeenshire	0.4	30000	330	623	4	7
Cumbria	0	35000	52219	109258	584	1145
Cumbria	0.05	30000	6643	20214	117	229
Cumbria	0.1	30000	6654	20426	118	231
Cumbria	0.15	30000	6683	20611	119	233
Cumbria	0.2	30000	6799	19959	115	226
Cumbria	0.4	30000	16473	48782	282	552
Devon	0	35000	804	7933	42	83
Devon	0.05	30000	354	1738	10	20
Devon	0.1	30000	316	1571	9	18
Devon	0.15	30000	271	1415	8	16
Devon	0.2	30000	364	1721	10	19
Devon	0.4	30000	206	1886	11	21
North Yorkshire	0	35000	61	111	1	1
North Yorkshire	0.05	30000	87	435	3	5
North Yorkshire	0.1	30000	62	113	1	1
North Yorkshire	0.15	30000	61	104	1	1
North Yorkshire	0.2	30000	61	104	1	1
North Yorkshire	0.4	30000	60	107	1	1

Culled cattle by daily farm cull capacity

County	Capacity (farms)	N	Mean culled cattle	sd	se	ci
Aberdeenshire	5	37000	455	860	4	9
Aberdeenshire	10	37000	472	886	5	9
Aberdeenshire	20	37000	483	932	5	9
Aberdeenshire	100	37000	492	967	5	10
Aberdeenshire	2925	37000	482	925	5	9
Cumbria	5	37000	26928	75925	395	774
Cumbria	10	37000	22214	67751	352	690
Cumbria	20	37000	16185	55053	286	561
Cumbria	100	37000	9500	34593	180	352
Cumbria	7884	37000	9638	35457	184	361
Devon	5	37000	539	5688	30	58
Devon	10	37000	308	2532	13	26
Devon	20	37000	234	2385	12	24
Devon	100	37000	383	1767	9	18
Devon	10662	37000	520	4844	25	49
North Yorkshire	5	37000	82	393	2	4
North Yorkshire	10	37000	61	103	1	1
North Yorkshire	20	37000	61	111	1	1
North Yorkshire	100	37000	62	112	1	1
North Yorkshire	7599	37000	62	111	1	1

Culled cattle by cull radius

County	Cull radius (km)	N	Mean culled cattle	sd	se	ci
Aberdeenshire	0	5000	274	508	7	14
Aberdeenshire	0.5	30000	304	546	3	6
Aberdeenshire	1	30000	367	650	4	7
Aberdeenshire	2	30000	486	861	5	10
Aberdeenshire	3	30000	542	991	6	11
Aberdeenshire	4	30000	576	1077	6	12
Aberdeenshire	5	30000	618	1197	7	14
Cumbria	0	5000	96388	138170	1954	3831
Cumbria	0.5	30000	16717	49924	288	565
Cumbria	1	30000	10596	40383	233	457
Cumbria	2	30000	10888	43779	253	495
Cumbria	3	30000	13110	49268	284	558
Cumbria	4	30000	15555	53287	308	603
Cumbria	5	30000	21244	64620	373	731
Devon	0	5000	1701	13218	187	366
Devon	0.5	30000	310	2846	16	32
Devon	1	30000	189	1648	10	19
Devon	2	30000	404	2482	14	28
Devon	3	30000	446	3278	19	37
Devon	4	30000	551	4772	28	54
Devon	5	30000	274	508	7	14
North Yorkshire	0	5000	304	546	3	6
North Yorkshire	0.5	30000	367	650	4	7
North Yorkshire	1	30000	486	861	5	10
North Yorkshire	2	30000	542	991	6	11

North Yorkshire	3		30000	576	1077	6	12
North Yorkshire	4		30000	618	1197	7	14
North Yorkshire	5		30000	96388	138170	1954	3831

Culled sheep by target density after culling

County	Density (farms/km^2)	N	Mean culled sheep	sd	se	ci
Aberdeenshire	0	35000	781	1773	9	19
Aberdeenshire	0.05	30000	938	2060	12	23
Aberdeenshire	0.1	30000	898	1951	11	22
Aberdeenshire	0.15	30000	832	1812	10	21
Aberdeenshire	0.2	30000	749	1643	9	19
Aberdeenshire	0.4	30000	526	1206	7	14
Cumbria	0	35000	208495	435041	2325	4558
Cumbria	0.05	30000	29312	91673	529	1037
Cumbria	0.1	30000	29382	92223	532	1044
Cumbria	0.15	30000	29197	92941	537	1052
Cumbria	0.2	30000	29370	89927	519	1018
Cumbria	0.4	30000	69723	208937	1206	2364
Devon	0	35000	2430	24290	130	254
Devon	0.05	30000	1157	6521	38	74
Devon	0.1	30000	1004	5718	33	65
Devon	0.15	30000	888	5263	30	60
Devon	0.2	30000	1181	6398	37	72
Devon	0.4	30000	671	6543	38	74
North Yorkshire	0	35000	335	624	3	7
North Yorkshire	0.05	30000	485	2860	17	32
North Yorkshire	0.1	30000	781	1773	9	19
North Yorkshire	0.15	30000	938	2060	12	23
North Yorkshire	0.2	30000	898	1951	11	22
North Yorkshire	0.4	30000	832	1812	10	21

Culled sheep by daily farm cull capacity

County	Capacity (farms)	N	Mean culled sheep	sd	se	ci
Aberdeenshire	5	37000	732	1663	9	17
Aberdeenshire	10	37000	775	1722	9	18
Aberdeenshire	20	37000	801	1798	9	18
Aberdeenshire	100	37000	822	1844	10	19
Aberdeenshire	2925	37000	808	1803	9	18
Cumbria	5	37000	110236	307187	1597	3130
Cumbria	10	37000	91029	273710	1423	2789
Cumbria	20	37000	66802	224156	1165	2284
Cumbria	100	37000	40025	142728	742	1454
Cumbria	7884	37000	40740	146580	762	1494
Devon	5	37000	1638	17429	91	178
Devon	10	37000	976	8377	44	85
Devon	20	37000	755	7849	41	80
Devon	100	37000	1256	6631	34	68
Devon	10662	37000	1648	15200	79	155
North Yorkshire	5	37000	447	2591	13	26
North Yorkshire	10	37000	344	656	3	7
North Yorkshire	20	37000	343	634	3	6
North Yorkshire	100	37000	347	667	3	7
North Yorkshire	7599	37000	342	627	3	6

Culled sheep by cull radius

County	Cull radius (km)	N	Mean culled sheep	sd	se	ci
Aberdeenshire	0	5000	410	954	13	26
Aberdeenshire	0.5	30000	483	1119	6	13
Aberdeenshire	1	30000	605	1347	8	15
Aberdeenshire	2	30000	807	1711	10	19
Aberdeenshire	3	30000	913	1936	11	22
Aberdeenshire	4	30000	950	2020	12	23
Aberdeenshire	5	30000	1030	2244	13	25
Cumbria	0	5000	381976	548483	7757	15207
Cumbria	0.5	30000	71790	212525	1227	2405
Cumbria	1	30000	45329	167500	967	1895
Cumbria	2	30000	45052	176291	1018	1995
Cumbria	3	30000	53772	197379	1140	2234
Cumbria	4	30000	63528	214994	1241	2433
Cumbria	5	30000	87092	263490	1521	2982
Devon	0	5000	4957	39435	558	1093
Devon	0.5	30000	998	9751	56	110
Devon	1	30000	621	5588	32	63
Devon	2	30000	1281	8419	49	95
Devon	3	30000	1432	10640	61	120
Devon	4	30000	1714	14842	86	168
Devon	5	30000	864	9175	53	104
North Yorkshire	0	5000	333	608	9	17
North Yorkshire	0.5	30000	473	2850	16	32
North Yorkshire	1	30000	344	640	4	7
North Yorkshire	2	30000	345	641	4	7

North Yorkshire	3	30000	343	666	4	8
North Yorkshire	4	30000	342	663	4	7
North Yorkshire	5	30000	346	670	4	8

Culled animals (culled cattle + culled sheep) by target density after culling

County	Density (farms/km^2)	N	Mean culled animals	sd	se	ci
Aberdeenshire	0	35000	1273	2601	14	27
Aberdeenshire	0.05	30000	1493	2985	17	34
Aberdeenshire	0.1	30000	1436	2858	17	32
Aberdeenshire	0.15	30000	1329	2627	15	30
Aberdeenshire	0.2	30000	1197	2372	14	27
Aberdeenshire	0.4	30000	856	1725	10	20
Cumbria	0	35000	260714	544173	2909	5701
Cumbria	0.05	30000	35955	111353	643	1260
Cumbria	0.1	30000	36036	112107	647	1269
Cumbria	0.15	30000	35880	113036	653	1279
Cumbria	0.2	30000	36169	109293	631	1237
Cumbria	0.4	30000	86195	257270	1485	2911
Devon	0	35000	3235	32163	172	337
Devon	0.05	30000	1511	8135	47	92
Devon	0.1	30000	1320	7189	42	81
Devon	0.15	30000	1158	6584	38	75
Devon	0.2	30000	1545	8008	46	91
Devon	0.4	30000	877	8384	48	95
North Yorkshire	0	35000	396	661	4	7
North Yorkshire	0.05	30000	572	3260	19	37
North Yorkshire	0.1	30000	408	701	4	8
North Yorkshire	0.15	30000	411	714	4	8
North Yorkshire	0.2	30000	400	677	4	8
North Yorkshire	0.4	30000	398	653	4	7

Culled animals (culled cattle + culled sheep) by daily farm cull capacity

County	Capacity (farms)	N	Mean culled animals	sd	se	ci
Aberdeenshire	5	37000	1186	2407	13	25
Aberdeenshire	10	37000	1247	2495	13	25
Aberdeenshire	20	37000	1284	2617	14	27
Aberdeenshire	100	37000	1313	2705	14	28
Aberdeenshire	2925	37000	1290	2613	14	27
Cumbria	5	37000	137165	382831	1990	3901
Cumbria	10	37000	113243	341197	1774	3477
Cumbria	20	37000	82987	278924	1450	2842
Cumbria	100	37000	49525	176939	920	1803
Cumbria	7884	37000	50377	181664	944	1851
Devon	5	37000	2177	23062	120	235
Devon	10	37000	1284	10837	56	110
Devon	20	37000	989	10175	53	104
Devon	100	37000	1639	8283	43	84
Devon	10662	37000	2169	19971	104	204
North Yorkshire	5	37000	529	2950	15	30
North Yorkshire	10	37000	404	690	4	7
North Yorkshire	20	37000	404	673	3	7
North Yorkshire	100	37000	409	704	4	7
North Yorkshire	7599	37000	403	662	3	7

Culled animals (culled cattle + culled sheep) by cull radius

County	Cull radius (km)	N	Mean culled animals	sd	se	ci
Aberdeenshire	0	5000	684	1374	19	38
Aberdeenshire	0.5	30000	788	1546	9	17
Aberdeenshire	1	30000	972	1870	11	21
Aberdeenshire	2	30000	1292	2446	14	28
Aberdeenshire	3	30000	1455	2804	16	32
Aberdeenshire	4	30000	1526	2988	17	34
Aberdeenshire	5	30000	1648	3336	19	38
Cumbria	0	5000	478364	686551	9709	19034
Cumbria	0.5	30000	88507	262005	1513	2965
Cumbria	1	30000	55925	207500	1198	2348
Cumbria	2	30000	55940	219808	1269	2487
Cumbria	3	30000	66882	246410	1423	2788
Cumbria	4	30000	79083	267983	1547	3033
Cumbria	5	30000	108336	327848	1893	3710
Devon	0	5000	6658	52599	744	1458
Devon	0.5	30000	1307	12541	72	142
Devon	1	30000	809	7189	42	81
Devon	2	30000	1686	10798	62	122
Devon	3	30000	1878	13827	80	156
Devon	4	30000	2266	19535	113	221
Devon	5	30000	1129	11886	69	135
North Yorkshire	0	5000	391	634	9	18
North Yorkshire	0.5	30000	558	3249	19	37
North Yorkshire	1	30000	405	671	4	8
North Yorkshire	2	30000	406	675	4	8
North Yorkshire	3	30000	404	703	4	8

North Yorkshire	4	30000	404	703	4	8
North Yorkshire	5	30000	408	714	4	8

Culled farms by target density after culling

County	Density (farms/km^2)	N	Mean culled farms	sd	se	ci
Aberdeenshire	0	35000	5	9	0	0
Aberdeenshire	0.05	30000	5	10	0	0
Aberdeenshire	0.1	30000	5	9	0	0
Aberdeenshire	0.15	30000	4	8	0	0
Aberdeenshire	0.2	30000	4	6	0	0
Aberdeenshire	0.4	30000	3	4	0	0
Cumbria	0	35000	416	847	5	9
Cumbria	0.05	30000	75	214	1	2
Cumbria	0.1	30000	72	212	1	2
Cumbria	0.15	30000	70	212	1	2
Cumbria	0.2	30000	68	204	1	2
Cumbria	0.4	30000	132	376	2	4
Devon	0	35000	9	82	0	1
Devon	0.05	30000	5	24	0	0
Devon	0.1	30000	4	20	0	0
Devon	0.15	30000	4	18	0	0
Devon	0.2	30000	4	20	0	0
Devon	0.4	30000	3	25	0	0
North Yorkshire	0	35000	1	0	0	0
North Yorkshire	0.05	30000	1	6	0	0
North Yorkshire	0.1	30000	1	1	0	0
North Yorkshire	0.15	30000	1	1	0	0
North Yorkshire	0.2	30000	1	1	0	0
North Yorkshire	0.4	30000	1	0	0	0

Culled farms by daily farm cull capacity

County	Capacity (farms)	N	Mean culled farms	sd	se	ci
Aberdeenshire	5	37000	4	7	0	0
Aberdeenshire	10	37000	4	8	0	0
Aberdeenshire	20	37000	4	8	0	0
Aberdeenshire	100	37000	5	9	0	0
Aberdeenshire	2925	37000	4	8	0	0
Cumbria	5	37000	224	603	3	6
Cumbria	10	37000	187	537	3	5
Cumbria	20	37000	141	444	2	5
Cumbria	100	37000	89	281	1	3
Cumbria	7884	37000	90	287	1	3
Devon	5	37000	6	51	0	1
Devon	10	37000	4	29	0	0
Devon	20	37000	3	35	0	0
Devon	100	37000	5	23	0	0
Devon	10662	37000	6	54	0	1
North Yorkshire	5	37000	1	5	0	0
North Yorkshire	10	37000	1	0	0	0
North Yorkshire	20	37000	1	0	0	0
North Yorkshire	100	37000	1	1	0	0
North Yorkshire	7599	37000	1	0	0	0

Culled farms by cull radius

County	Cull radius (km)	N	Mean culled farms	sd	se	ci
Aberdeenshire	0	5000	2	3	0	0
Aberdeenshire	0.5	30000	3	4	0	0
Aberdeenshire	1	30000	4	6	0	0
Aberdeenshire	2	30000	5	8	0	0
Aberdeenshire	3	30000	5	9	0	0
Aberdeenshire	4	30000	5	9	0	0
Aberdeenshire	5	30000	5	10	0	0
Cumbria	0	5000	738	1058	15	29
Cumbria	0.5	30000	167	464	3	5
Cumbria	1	30000	105	347	2	4
Cumbria	2	30000	99	350	2	4
Cumbria	3	30000	113	386	2	4
Cumbria	4	30000	127	411	2	5
Cumbria	5	30000	168	487	3	6
Devon	0	5000	15	115	2	3
Devon	0.5	30000	4	43	0	0
Devon	1	30000	3	23	0	0
Devon	2	30000	5	29	0	0
Devon	3	30000	5	32	0	0
Devon	4	30000	6	45	0	1
Devon	5	30000	3	41	0	0
North Yorkshire	0	5000	1	0	0	0
North Yorkshire	0.5	30000	1	6	0	0
North Yorkshire	1	30000	1	0	0	0
North Yorkshire	2	30000	1	0	0	0
North Yorkshire	3	30000	1	1	0	0

North Yorkshire	4	30000	1	1	0	0
North Yorkshire	5	30000	1	1	0	0

Epidemic length (days) by target density after culling

County	Density (farms/km^2)	N	Mean epidemic length (days)	sd	se	ci
Aberdeenshire	0	35000	19	12	0	0
Aberdeenshire	0.05	30000	18	10	0	0
Aberdeenshire	0.1	30000	19	10	0	0
Aberdeenshire	0.15	30000	19	11	0	0
Aberdeenshire	0.2	30000	19	11	0	0
Aberdeenshire	0.4	30000	20	13	0	0
Cumbria	0	35000	110	177	1	2
Cumbria	0.05	30000	40	56	0	1
Cumbria	0.1	30000	41	56	0	1
Cumbria	0.15	30000	41	56	0	1
Cumbria	0.2	30000	42	57	0	1
Cumbria	0.4	30000	69	113	1	1
Devon	0	35000	18	39	0	0
Devon	0.05	30000	15	10	0	0
Devon	0.1	30000	15	10	0	0
Devon	0.15	30000	14	8	0	0
Devon	0.2	30000	15	10	0	0
Devon	0.4	30000	14	10	0	0
North Yorkshire	0	35000	13	0	0	0
North Yorkshire	0.05	30000	14	6	0	0
North Yorkshire	0.1	30000	13	0	0	0
North Yorkshire	0.15	30000	13	1	0	0
North Yorkshire	0.2	30000	13	0	0	0
North Yorkshire	0.4	30000	13	0	0	0

Epidemic length (days) by daily farm cull capacity

County	Capacity (farms)	N	Mean epidemic length (days)	sd	se	ci
Aberdeenshire	5	37000	19	12	0	0
Aberdeenshire	10	37000	19	11	0	0
Aberdeenshire	20	37000	19	11	0	0
Aberdeenshire	100	37000	19	11	0	0
Aberdeenshire	2925	37000	19	11	0	0
Cumbria	5	37000	75	132	1	1
Cumbria	10	37000	67	119	1	1
Cumbria	20	37000	57	99	1	1
Cumbria	100	37000	47	76	0	1
Cumbria	7884	37000	47	77	0	1
Devon	5	37000	16	30	0	0
Devon	10	37000	15	15	0	0
Devon	20	37000	14	8	0	0
Devon	100	37000	16	12	0	0
Devon	10662	37000	16	22	0	0
North Yorkshire	5	37000	13	5	0	0
North Yorkshire	10	37000	13	0	0	0
North Yorkshire	20	37000	13	0	0	0
North Yorkshire	100	37000	13	0	0	0
North Yorkshire	7599	37000	13	0	0	0

Epidemic length (days) by cull radius

County	Cull radius (km)	N	Mean epidemic length (days)	sd	se	ci
Aberdeenshire	0	5000	21	16	0	0
Aberdeenshire	0.5	30000	19	12	0	0
Aberdeenshire	1	30000	19	11	0	0
Aberdeenshire	2	30000	19	11	0	0
Aberdeenshire	3	30000	19	11	0	0
Aberdeenshire	4	30000	19	11	0	0
Aberdeenshire	5	30000	19	12	0	0
Cumbria	0	5000	183	232	3	6
Cumbria	0.5	30000	72	112	1	1
Cumbria	1	30000	52	78	0	1
Cumbria	2	30000	45	74	0	1
Cumbria	3	30000	47	83	0	1
Cumbria	4	30000	52	93	1	1
Cumbria	5	30000	62	120	1	1
Devon	0	5000	24	66	1	2
Devon	0.5	30000	16	17	0	0
Devon	1	30000	14	9	0	0
Devon	2	30000	16	14	0	0
Devon	3	30000	15	17	0	0
Devon	4	30000	16	25	0	0
Devon	5	30000	14	6	0	0
North Yorkshire	0	5000	13	0	0	0
North Yorkshire	0.5	30000	14	6	0	0
North Yorkshire	1	30000	13	0	0	0
North Yorkshire	2	30000	13	0	0	0
North Yorkshire	3	30000	13	1	0	0

North Yorkshire	4	30000	13		1	0	0
North Yorkshire	5	30000	13		0	0	0
