# Supplementary material for: Modeling target-density-based cull strategies to contain foot-and-mouth disease outbreaks
Source: PeerJ. 2024 Feb 29;12:e16998. doi: 10.7717/peerj.16998 (PMC10909358; doi:10.7717/peerj.16998)
Supplement: Supplemental Information 9 — * = P < 0.05, ** = P < 0.01, and *** = P < 0.001. [file peerj-12-16998-s009.pdf]

Target farm density and culled cattle

County	Coefficient	Estimate	Standard error	t-value	Pr(>|t|)
Aberdeenshire	Intercept	476.26	14.07	33.855	5.67e-05 ***
Aberdeenshire	Linear term	-161.87	34.46	-4.698	0.0182 *
Aberdeenshire	Quadratic term	-54.76	34.46	-1.589	0.2102
Cumbria	Intercept	15912	5554	2.865	0.0643
Cumbria	Linear term	-13825	13605	-1.016	0.3844
Cumbria	Quadratic term	30198	13605	2.220	0.1131
Devon	Intercept	385.67	62.66	6.155	0.00863 **
Devon	Linear term	-323.33	153.49	-2.107	0.12578
Devon	Quadratic term	228.37	153.49	1.488	0.23350
North Yorkshire	Intercept	65.516	5.250	12.479	0.00111 **
North Yorkshire	Linear term	-8.830	12.860	-0.687	0.54163
North Yorkshire	Quadratic term	2.146	12.860	0.167	0.87810

Target farm density and culled sheep

County	Coefficient	Estimate	Standard error	t-value	Pr(>|t|)
Aberdeenshire	Intercept	787.59	29.36	26.826	0.000114 ***
Aberdeenshire	Linear term	-274.63	71.92	-3.819	0.031598 *
Aberdeenshire	Quadratic term	-125.56	71.92	-1.746	0.179156
Cumbria	Intercept	65913	21748	3.031	0.0563
Cumbria	Linear term	-53048	53272	-0.996	0.3927
Cumbria	Quadratic term	119892	53272	2.251	0.1099
Devon	Intercept	1221.9	181.7	6.724	0.00672 **
Devon	Linear term	-960.2	445.1	-2.157	0.11991
Devon	Quadratic term	642.0	445.1	1.442	0.24491
North Yorkshire	Intercept	365.327	28.884	12.648	0.00107 **
North Yorkshire	Linear term	-47.139	70.750	-0.666	0.55290
North Yorkshire	Quadratic term	8.161	70.750	0.115	0.91545

Target farm density and culled animals

County	Coefficient	Estimate	Standard error	t-value	Pr(>|t|)
Aberdeenshire	Intercept	1263.85	43.36	29.148	8.87e-05 ***
Aberdeenshire	Linear term	-436.50	106.21	-4.110	0.0261 *
Aberdeenshire	Quadratic term	-180.32	106.21	-1.698	0.1881
Cumbria	Intercept	81825	27302	2.997	0.0578
Cumbria	Linear term	-66873	66877	-1.000	0.3910
Cumbria	Quadratic term	150090	66877	2.244	0.1105
Devon	Intercept	1607.6	244.3	6.580	0.00714 **
Devon	Linear term	-1283.5	598.5	-2.145	0.12132
Devon	Quadratic term	870.4	598.5	1.454	0.24184
North Yorkshire	Intercept	430.84	34.12	12.628	0.00107 **
North Yorkshire	Linear term	-55.97	83.57	-0.670	0.55098
North Yorkshire	Quadratic term	10.31	83.57	0.123	0.90964

Target farm density and culled farms

County	Coefficient	Estimate	Standard error	t-value	Pr(>|t|)
Aberdeenshire	Intercept	4.3500	0.1693	25.688	0.000129 ***
Aberdeenshire	Linear term	-2.0433	0.4148	-4.926	0.016035 *
Aberdeenshire	Quadratic term	-0.1091	0.4148	-0.263	0.809553
Cumbria	Intercept	138.78	41.19	3.369	0.0434 *
Cumbria	Linear term	-117.60	100.89	-1.66	0.3280
Cumbria	Quadratic term	225.63	100.89	2.236	0.1113
Devon	Intercept	4.8043	0.5038	9.536	0.00245 **
Devon	Linear term	-3.7239	1.2341	-3.017	0.05688
Devon	Quadratic term	2.1173	1.2341	1.716	0.18474
North Yorkshire	Intercept	1.07564	0.07095	15.160	0.000623 ***
North Yorkshire	Linear term	-0.11922	0.17380	-0.686	0.541992
North Yorkshire	Quadratic term	0.03439	0.17380	0.198	0.855792

Target farm density and epidemic length

County	Coefficient	Estimate	Standard error	t-value	Pr(>|t|)
Aberdeenshire	Intercept	19.1389	0.1558	122.876	1.19e-06 ***
Aberdeenshire	Linear term	0.8170	0.3815	2.141	0.122
Aberdeenshire	Quadratic term	0.5251	0.3815	1.376	0.262
Cumbria	Intercept	57.133	8.359	6.835	0.00641 **
Cumbria	Linear term	-10.504	20.476	-0.513	0.64334
Cumbria	Quadratic term	50.827	20.476	2.482	0.08909
Devon	Intercept	15.4333	0.4471	34.521	5.34e-05 ***
Devon	Linear term	-2.1477	1.0951	-1.961	0.145
Devon	Quadratic term	1.8741	1.0951	1.711	0.186
North Yorkshire	Intercept	13.1044	0.1001	130.929	9.82e-07 ***
North Yorkshire	Linear term	-0.1590	0.2452	-0.649	0.563
North Yorkshire	Quadratic term	0.0565	0.2452	0.230	0.833

Daily farm cull capacity and culled cattle

County	Coefficient	Estimate	Standard error	t-value	Pr(>|t|)
Aberdeenshire	Intercept	476.671	5.505	86.592	0.000133 ***
Aberdeenshire	Linear term	7.053	12.309	0.573	0.624474
Aberdeenshire	Quadratic term	-21.362	12.309	-1.735	0.224788
Cumbria	Intercept	16893	1914	8.827	0.0126 *
Cumbria	Linear term	-8239	4279	-1.925	0.1941
Cumbria	Quadratic term	11503	4279	2.688	0.1150
Devon	Intercept	396.99	71.27	5.570	0.0308 *
Devon	Linear term	137.97	159.37	0.866	0.4779
Devon	Quadratic term	10.46	159.37	0.066	0.9536
North Yorkshire	Intercept	65.388	5.142	12.716	0.00613 **
North Yorkshire	Linear term	-4.260	11.498	-0.370	0.74657
North Yorkshire	Quadratic term	7.221	11.498	0.628	0.59415

Daily farm cull capacity and culled sheep

County	Coefficient	Estimate	Standard error	t-value	Pr(>|t|)
Aberdeenshire	Intercept	787.41	13.55	58.108	0.000296 ***
Aberdeenshire	Linear term	24.15	30.30	0.797	0.509072
Aberdeenshire	Quadratic term	-51.07	30.30	-1.685	0.233953
Cumbria	Intercept	69766	7747	9.005	0.0121 *
Cumbria	Linear term	-32963	17323	-1.903	0.1974
Cumbria	Quadratic term	46249	17323	2.670	0.1163
Devon	Intercept	1254.59	208.56	6.016	0.0265 *
Devon	Linear term	440.25	466.35	0.944	0.4448
Devon	Quadratic term	-28.92	466.35	-0.062	0.9562
North Yorkshire	Intercept	364.52	25.67	14.200	0.00492 **
North Yorkshire	Linear term	-26.02	57.40	-0.453	0.69476
North Yorkshire	Quadratic term	34.95	57.40	0.609	0.60450

Daily farm cull capacity and culled animals

County	Coefficient	Estimate	Standard error	t-value	Pr(>|t|)
Aberdeenshire	Intercept	1264.08	19.05	66.359	0.000227 ***
Aberdeenshire	Linear term	31.20	42.60	0.732	0.540085
Aberdeenshire	Quadratic term	-72.43	42.60	-1.700	0.231151
Cumbria	Intercept	86660	9661	8.970	0.0122 *
Cumbria	Linear term	-41202	21602	-1.907	0.1967
Cumbria	Quadratic term	57753	21602	2.673	0.1161
Devon	Intercept	1651.58	279.83	5.902	0.0275 *
Devon	Linear term	578.22	625.71	0.924	0.4530
Devon	Quadratic term	-18.46	625.71	-0.029	0.9791
North Yorkshire	Intercept	429.91	30.81	13.953	0.0051 **
North Yorkshire	Linear term	-30.28	68.89	-0.440	0.7032
North Yorkshire	Quadratic term	42.18	68.89	0.612	0.6027

Daily farm cull capacity and culled farms

County	Coefficient	Estimate	Standard error	t-value	Pr(>|t|)
Aberdeenshire	Intercept	4.36416	0.07573	57.625	0.000301 ***
Aberdeenshire	Linear term	0.15465	0.16935	0.913	0.457534
Aberdeenshire	Quadratic term	-0.28272	0.16935	-1.669	0.236974
Cumbria	Intercept	146.28	14.69	9.957	0.00994 **
Cumbria	Linear term	-64.07	32.85	-1.950	0.19042
Cumbria	Quadratic term	89.27	32.85	2.718	0.11292
Devon	Intercept	4.9127	0.6522	7.532	0.0172 *
Devon	Linear term	1.5908	1.4584	1.091	0.3893
Devon	Quadratic term	-0.5102	1.4584	-0.350	0.7599
North Yorkshire	Intercept	1.07402	0.07179	14.962	0.00444 **
North Yorkshire	Linear term	-0.06741	0.16052	-0.420	0.71535
North Yorkshire	Quadratic term	0.10291	0.16052	0.641	0.58710

Daily farm cull capacity and epidemic length

County	Coefficient	Estimate	Standard error	t-value	Pr(>|t|)
Aberdeenshire	Intercept	19.14605	0.03402	562.751	3.16e-06 ***
Aberdeenshire	Linear term	-0.11385	0.07608	-1.497	0.273
Aberdeenshire	Quadratic term	0.17971	0.07608	2.362	0.142
Cumbria	Intercept	58.560	3.053	19.182	0.00271 **
Cumbria	Linear term	-13.231	6.827	-1.938	0.19218
Cumbria	Quadratic term	18.209	6.827	2.667	0.11649
Devon	Intercept	15.5158	0.4881	31.788	0.000988 ***
Devon	Linear term	0.5149	1.0914	0.472	0.683571
Devon	Quadratic term	-0.2388	1.0914	-0.219	0.847091
North Yorkshire	Intercept	13.10217	0.10068	130.138	5.9e-05 ***
North Yorkshire	Linear term	-0.09452	0.22512	-0.420	0.715
North Yorkshire	Quadratic term	0.14942	0.22512	0.664	0.575

Cull radius and culled cattle

County	Coefficient	Estimate	Standard error	t-value	Pr(>|t|)
Aberdeenshire	Intercept	452.565	5.916	76.502	1.75e-17 ***
Aberdeenshire	Linear term	327.296	15.651	20.912	3.09e-05 ***
Aberdeenshire	Quadratic term	-68.621	15.651	-4.384	0.0118 *
Cumbria	Intercept	26357	9328	2.825	0.0476 *
Cumbria	Linear term	-34981	24680	-1.417	0.2293
Cumbria	Quadratic term	46295	24680	1.876	0.1339
Devon	Intercept	552.3	192.1	2.876	0.0452 *
Devon	Linear term	-555.4	508.1	-1.093	0.3358
Devon	Quadratic term	535.0	508.1	1.053	0.3518
North Yorkshire	Intercept	64.595	4.122	15.670	9.69e-05 ***
North Yorkshire	Linear term	-7.034	10.906	-0.645	0.554
North Yorkshire	Quadratic term	2.358	10.906	0.216	0.839

Cull radius and culled sheep

County	Coefficient	Estimate	Standard error	t-value	Pr(>|t|)
Aberdeenshire	Intercept	742.44	10.23	72.568	2.16e-07 ***
Aberdeenshire	Linear term	576.93	27.07	21.314	2.87e-05 ***
Aberdeenshire	Quadratic term	-170.88	27.07	-5.205	0.00649 **
Cumbria	Intercept	106934	36204	2.954	0.0418 *
Cumbria	Linear term	-138863	95788	-1.450	0.2207
Cumbria	Quadratic term	183379	95788	1.914	0.1281
Devon	Intercept	1695.3	552.9	3.066	0.0374 *
Devon	Linear term	-1556.3	1462.8	-1.064	0.3473
Devon	Quadratic term	1492.8	1462.8	1.020	0.3652
North Yorkshire	Intercept	360.71	21.36	16.886	7.21e-05 ***
North Yorkshire	Linear term	-42.44	56.52	-0.751	0.494
North Yorkshire	Quadratic term	11.88	56.52	0.210	0.844

Cull radius and culled animals

County	Coefficient	Estimate	Standard error	t-value	Pr(>|t|)
Aberdeenshire	Intercept	1195.00	15.89	75.186	1.88e-07 ***
Aberdeenshire	Linear term	904.23	42.05	21.503	2.77e-05 ***
Aberdeenshire	Quadratic term	-209.51	42.05	-4.982	0.00759 **
Cumbria	Intercept	133291	45533	2.927	0.0429 *
Cumbria	Linear term	-173843	120468	-1.443	0.2225
Cumbria	Quadratic term	229674	120468	1.907	0.1293
Devon	Intercept	2247.6	744.9	3.017	0.0939 *
Devon	Linear term	-2111.7	1970.9	-1.071	0.3443
Devon	Quadratic term	2027.8	1970.9	1.029	0.3617
North Yorkshire	Intercept	425.31	25.48	16.692	7.55e-05 ***
North Yorkshire	Linear term	-49.47	67.41	-0.734	0.504
North Yorkshire	Quadratic term	14.24	67.41	0.211	0.843

Cull radius and culled farms

County	Coefficient	Estimate	Standard error	t-value	Pr(>|t|)
Aberdeenshire	Intercept	4.10233	0.07631	53.758	7.17e-07 ***
Aberdeenshire	Linear term	2.77121	0.20190	13.726	0.000163 ***
Aberdeenshire	Quadratic term	-1.08836	0.20190	-5.391	0.005728 **
Cumbria	Intercept	216.75	66.53	3.258	0.0311 *
Cumbria	Linear term	-281.10	176.02	-1.597	0.1855
Cumbria	Quadratic term	344.92	176.02	1.960	0.1216
Devon	Intercept	6.142	1.600	3.838	0.0185 *
Devon	Linear term	-4.442	4.234	-1.049	0.3533
Devon	Quadratic term	3.791	4.234	0.895	0.4212
North Yorkshire	Intercept	1.06540	0.05641	18.885	4.63e-05 ***
North Yorkshire	Linear term	-0.11789	0.14926	-0.790	0.474
North Yorkshire	Quadratic term	0.03594	0.14926	0.241	0.822

Cull radius and epidemic length

County	Coefficient	Estimate	Standard error	t-value	Pr(>|t|)
Aberdeenshire	Intercept	19.3774	0.2035	95.205	7.3e-08 ***
Aberdeenshire	Linear term	-0.9575	0.5385	-1.778	0.1500
Aberdeenshire	Quadratic term	1.2082	0.5385	2.244	0.0883
Cumbria	Intercept	73.39	12.53	5.858	0.00424 **
Cumbria	Linear term	-65.71	33.14	-1.983	0.11844
Cumbria	Quadratic term	76.55	33.14	2.310	0.08206
Devon	Intercept	16.532	1.096	15.086	0.000113 ***
Devon	Linear term	-5.087	2.899	-1.755	0.154181
Devon	Quadratic term	3.370	2.899	1.162	0.309709
North Yorkshire	Intercept	13.09180	0.08042	162.797	8.54e-09 ***
North Yorkshire	Linear term	-0.18828	0.21277	-0.885	0.426
North Yorkshire	Quadratic term	0.05781	0.21277	0.272	0.799
