# Supplementary material for: Modeling target-density-based cull strategies to contain foot-and-mouth disease outbreaks
Source: PeerJ. 2024 Feb 29;12:e16998. doi: 10.7717/peerj.16998 (PMC10909358; doi:10.7717/peerj.16998)
Supplement: Supplemental Information 10 — Percentages of total simulations outbreaks are indicated in parentheses. [file peerj-12-16998-s010.pdf]

Effect of cull radius averaged over all daily farm cull capacities

County	Cull radius (km)	Outbreaks where culled farms >1
Aberdeenshire	0	1712 (34.2)
Aberdeenshire	0.5	9868 (32.9)
Aberdeenshire	1	9971 (33.2)
Aberdeenshire	2	10129 (33.8)
Aberdeenshire	3	10084 (33.6)
Aberdeenshire	4	10066 (33.6)
Aberdeenshire	5	10093 (33.6)
Cumbria	0	2933 (58.7)
Cumbria	0.5	17826 (59.4)
Cumbria	1	17712 (59.0)
Cumbria	2	17672 (58.9)
Cumbria	3	17870 (59.6)
Cumbria	4	17899 (59.7)
Cumbria	5	17997 (60.0)
Devon	0	2512 (50.2)
Devon	0.5	14883 (49.6)
Devon	1	14746 (49.2)
Devon	2	14719 (49.1)
Devon	3	14736 (49.1)
Devon	4	14845 (49.5)
Devon	5	14844 (49.5)
North Yorkshire	0	2403 (48.1)
North Yorkshire	0.5	14467 (48.2)
North Yorkshire	1	14769 (49.2)
North Yorkshire	2	14649 (48.8)
North Yorkshire	3	14622 (48.7)
North Yorkshire	4	14589 (48.6)
North Yorkshire	5	14379 (47.9)

Effect of daily farm cull capacity averaged over all cull radii

County	Capacity (farms/day)	Outbreaks where culled farms > 1
Aberdeenshire	5	12247 (33.1)
Aberdeenshire	10	12441 (33.62)
Aberdeenshire	20	12476 (33.72)
Aberdeenshire	100	12361 (33.41)
Aberdeenshire	Unlimited	12398 (33.51)
Cumbria	5	22076 (59.66)
Cumbria	10	21887 (59.15)
Cumbria	20	22063 (59.63)
Cumbria	100	21975 (59.39)
Cumbria	Unlimited	21908 (59.21)_
Devon	5	18305 (49.47)
Devon	10	18139 (49.02)
Devon	20	18391 (49.71)
Devon	100	18293 (49.44)
Devon	Unlimited	18157 (49.07)
North Yorkshire	5	18068 (48.83)
North Yorkshire	10	17938 (48.48)
North Yorkshire	20	18048 (48.78)
North Yorkshire	100	17806 (48.12)
North Yorkshire	Unlimited	18018 (48.7)
